# Depressive Symptoms and One Year Mortality among Elderly Patients Discharged from a Rehabilitation Ward after Orthopaedic Surgery of the Lower Limbs

**DOI:** 10.3233/BEN-2010-0274

**Published:** 2010-11-23

**Authors:** Fabio Guerini, Sara Morghen, Elena Lucchi, Giuseppe Bellelli, Marco Trabucchi

**Affiliations:** ^1^Department of Rehabilitation and Aged Care “Ancelle della Carità” HospitalCremonaItaly; ^2^Geriatric Research Group BresciaBresciaItaly; ^3^University Tor VergataRome and Geriatric Research Group BresciaBresciaItaly

**Keywords:** Depressive symptoms, mortality, orthopaedic rehabilitation, elderly

## Abstract

The objective of the present prospective observational study is to evaluate the effect of depressive symptoms on 1-year mortality in a population of elderly patients discharged from a rehabilitation unit after orthopaedic surgery of the lower limbs. A total of 222 elderly inpatients were included, and stratified according to 12-months survival. 14 (6.3%) of the patients who were eligible for this study died during the 12-months period after discharge. As expected, patients who died were significantly older, lower cognitive performance, more depressive symptoms, poorer nutritional status and higher comorbidity in comparison to those who survived. Furthermore, they were generally more functionally dependent on admission to the Department, had worse functional recovery and were more disable at discharge, although a longer length of stay comparing to survived patients. In the adjusted logistic regression model, after adjustment for possible confounders and covariates, the presence of severe depressive symptoms significantly predicted a four-fold risk of death at 12 months. The only other factor associated poor 12-months survival was comorbidity, that predicted a 6-fold risk of death. In conclusions this study suggests that severe depressive symptoms on admission predicts 1-year mortality in elderly patients discharged from a post-acute care unit after orthopaedic rehabilitation.

## 1. Introduction

Depressive symptoms are common among older medical inpatients [1]. Several studies have shown that DS are a risk factor for adverse outcomes and mortality in selected populations of patients, including those with stroke and cardiovascular diseases [2,3]. However, few studies systematically evaluated the impact of depressive symptoms in orthopaedic patients, especially after arthroplastic surgery. Nightingale et al. found that depressive symptoms increase the risk of mortality at 2 years in patients after hip fracture [4]. On the contrary Holmes and House found that the relative risks of mortality over 6 months after hip fracture was increased in dementia and delirium, but not in depressed patients [5]. More recently Herschkovitz et al. showed that only dementia and age were independent predictors of mortality during the first 2 years after discharge from a post-acute rehabilitation program, while the presence of depression was not [6].

This apparent inconsistency of results may be due to the fact that these studies did not report the severity of depressive symptoms, but only whether the patient were positive to depressive symptoms screening or not [5, 6]. Another confounding effect may be due to inadequate adjustment for other risk factors for mortality, such as comorbidity, physical disability, and cognitive impairment.

ISSN 0953-4180/10/$27.50 © 2010 – IOS Press and the authors. All rights reserved

The aim of this study is to evaluate the effect of depressive symptoms severity on 1-year mortality in a population of elderly patients discharged from a rehabilitation unit after orthopaedic surgery of the lower limbs. This effect was measured taking into the account for the possible interference of socio-demographic, clinical, cognitive and functional variables.

## 2. Methods

### 2.1. Setting

The Department of Rehabilitation and Aged Care is a 80-beds ward devoted to the rehabilitation of post-acute and chronic disabilities of elderly patients. The most frequent reasons for admission are post-surgical interventions (hip fracture surgical repair, hip or knee arthroplasty, abdominal, cardiac or thoracic surgery), stroke (recent or chronic), peripheral vascular diseases, subacute and chronic heart failure, subacute and chronic obstructive pulmonary diseases, Parkinson diseases and parkinsonisms, gait and balance disorders due to a single or mixed etiology, including hypokinetic syndrome [7].

### 2.2. Subjects

The study sample was taken from all new and consecutive admissions to our Department from January 1st, 2004 to May 31st, 2007. Inclusion criteria were age greater or equal to 65 years and the need of rehabilitation after orthopaedic surgery (hip fracture surgical repair, elective knee or hip surgical replacement). Exclusion criteria were a length of stay in the orthopaedic ward before admission longer than 1 week, a written order not to ambulate after orthopaedic surgery, pathological or multiple fractures and/or other illnesses reducing life expectancy to less than 6 months or poor reliability of self reported depressive symptoms (score $<$ 15/30 on Mini Mental State Examination – MMSE) [8].

Informed consent was obtained on admission by the patients or their legal representative. The study was approved by the Ethics Committee of Gerontological Sciences of the Geriatric Research Group.

### 2.3. Comprehensive geriatric assessment

All subjects underwent on admission a comprehensive multidimensional geriatric assessment including demographics, cognitive, clinical and functional characteristics. The global cognitive status was assessed with the Mini Mental State Examination (MMSE), and the physical health status with the Charlson Index, a well-known measure of comorbidity [9]. Depressive symptoms were evaluated on admission using the 15-items Geriatric Depression Scale (GDS), a commonly used screening tool in geriatric settings, with higher scores indicating worse affective status [10]. According to previous studies, patients were defined as having mild to moderate depressive symptoms when the GDS score ranged from 6 to 10/15, while having severe depressive symptoms when GDS score was greater or equal to 11/15 [10,11]. The functional status was assessed both on admission and at discharge using the Functional Independence Measure (FIM) [12].

### 2.4. Follow-up

At 12 months, deaths were investigated with a telephone follow-up interview with patients (or proxies living with patients for those with cognitive impairment or in case of death) by a psychologist trained in geriatrics and blinded to the aim of this study.

### 2.5. Statistical analysis

All analyses were performed using the SPSS (Statistical Package for Social Sciences) software tool, version 11.0. Significance between variables was tested with the GLM model, or chisquare analysis, when appropriate. The independent association of death at 1 year with possible predictors was tested in multiple logistic regression model (method stepwise), with covariates and confounders treated as categorical variables (age, gender, GDS, MMSE, FIM, Charlson index, orthopaedic categories). For continuous variables that entered in the model, an analysis of the quartiles has been performed in order to select the best cut-off to categorize these variables.

## 3. Results

In Table 1 are shown the demographic, clinical and functional characteristics of 222 patients who were eligible for this study. Of these, 14 (6.3%) died during the 12-months period after discharge. As expected, patients who died were significantly older, had lower cognitive performance and more severe depressive symptoms. From a clinical point of view they had more frequently hip fracture, poorer nutritional status (as ex-

Table 1
Demographic, clinical and functional characteristics of 222 orthopaedic elderly patients admitted to the Department of Rehabilitation and Aged Care according to 12-months survival

| | Death ($n$ = 14, 6.3%) Mean ± SD or n(%) | Alive ($n$ = 208, 93.7%) Mean ± SD or n(%) | P |
|---|---|---|---|
| Demographics | | | |
| Age, years | 82.2 ± 6.5[c] | 77.4 ± 7.7 | 0.000 |
| Gender female | 11 (78.6%) | 164 (78.8%) | N.S. |
| Orthopaedic categories | | | 0.037 |
| Hip fracture surgical repair | 12 (85.7%) | 122 (58.7%) | |
| Elective knee or hip replacement | 2 (14.3%) | 86 (41.3%) | |
| Cognitive and affective status | | | |
| Mini Mental State Examination (0–30) | 22.3 ± 4.0 | 25.4 ± 3.7 | 0.003 |
| Geriatric Depression Scale (0–15) | 7.0 ± 3.1 | 4.5 ± 3.1 | 0.005 |
| − GDS $\leqslant$ 5/15 | 4 (28.6%) | 141 (67.8%) | |
| − GDS from 6 to 10/15 | 7 (50.0%) | 57 (27.4%) | |
| − GDS $\geqslant$ 11/15 | 3 (21.4%) | 10 (4.8%) | |
| Health Status | | | |
| Charlson index of comorbidity (0–37) | 4.1 ± 2.6 | 1.8 ± 1.8 | < 0.001 |
| Serum albumin levels (gr/dl) | 2.6 ± 0.4 | 2.9 ± 0.4 | 0.004 |
| Serum cholesterol levels (gr/dl) | 134.1 ± 36.9 | 167.9 ± 35.3 | 0.001 |
| Functional Status | | | |
| FIM* Admission (0–126) | 56.0 ± 18.3 | 75.6 ± 21.8 | 0.001 |
| FIM* Discharge (0–126) | 72.0 ± 26.8 | 99.5 ± 19.6 | < 0.001 |
| Length of Stay, days | 30.1 ± 9.4 | 24.2 ± 7.9 | 0.008 |

$P$ denotes significance between variables using the GLM model (or chi-square analysis where appropriate).
*FIM = Functional Independence Measure.

Table 2
Predictors of 12-months mortality in a population of 222 orthopaedic elderly patients admitted to the Department of Rehabilitation and Aged Care in multiple logistic regression model

| | OR (Confidence intervals, 95%) | p |
|---|---|---|
| Severe depressive symptoms | 4.4 (1.4 to 14.0) | 0.013 |
| Moderate to severe comorbidity | 6.0 (1.8 to 20.2) | 0.004 |

Severe depressive symptoms: Geriatric Depression Scale $\geqslant$ 11/15. Moderate to severe comorbidity: Charlson index > 2.
OR denotes odds ratio; 95% CI denotes Confidence Intervals and p-value the associated significance computed in adjusted regression model. Variables not entered in the model were: age, gender, orthopaedic categories, and categorized GDS, MMSE, FIM.

pressed from serum albumin and cholesterol levels) and higher comorbidity (number of diseases at Charlson index) in comparison to those who survived. Furthermore, they were generally more functionally dependent on admission to the Department, and although a longer length of stay they were more disable at discharge comparing to survived patients (as expressed at the FIM score).

The adjusted regression model assessing the power of depressive symptoms and other covariates (treated as categorical variables) to predict deaths at follow-up is shown in Table 2. It could be observed that the presence of severe depressive symptoms predicted a four-fold risk of death. Interestingly, the only other factor associated with 12-months deaths was comorbidity (measured with the Charlson index), predicting a 6-fold risk of death. The other covariates in the model (age, gender, GDS, MMSE, FIM, orthopaedic categories) were not significant predictors.

## 4. Discussion

The study shows that severe depressive symptoms are predictors, in addition to comorbidity, of 1-year mortality in post-surgery orthopaedic rehabilitation. The association is independent from a number of potential clinical, cognitive and functional confounders.

Depressive symptoms in old patients are known to have a large clinical heterogeneity, which may exert these adverse effects throughout different mechanisms. On the one side, the lack of motivation might affect the participation of patient with depressive symptoms in the rehabilitative project [13], which in turn might influence the functional recovery at discharge and the adherence to pharmacological treatments. This could lead to inadequate lifestyle habits and increase the susceptibility to diseases. On the other side, depressive symptoms may be viewed as a marker of frailty, indirectly revealing the lack of patient's competence toward distressful events. In line with this observation, recent studies have shown a derangement of some biochemical circuits (serotonine and noradrenaline transmitters) [14] and an increase of atherosclerosis in depressed patients [15].

The observation that comorbidity is an independent predictor of mortality in elderly patients after orthopaedic surgical intervention is not surprising, since it has been demonstrated in several studies [16,17].

However, the finding that only severe depressive symptoms, and not mild or moderate depressive symptoms, are associated with 1-year mortality deserves comments. The most probable explanation is that high scores on GDS are more accurate to detect a real affective disorder or an underlying condition of frailty, which in turn may lead to increased mortality. In line with this interpretation, a study by Chiang and colleagues has recently shown that the 15-items GDS is more accurate to detect moderate or severe levels of depression than mild ones [18].

Our data are in line to previous studies showing an inverse relationship between severity of depressive symptoms and adverse clinical outcomes [19] and suggest that physicians may consider these symptoms as a key target of their routine rehabilitative practice.

A limitation of this study is that our population included patients with elective and non-elective surgical interventions. However, it should be underscored that in the multiple logistic regression model we considered this confounding factor, and the orthopaedic typology (hip fracture surgical repair vs elective knee or hip surgical replacement) did not significantly predict mortality in this population. Another limitation is the population size, thus further studies are needed, with higher number of cases, in particular on patients with severe depressive symptoms, to confirm our findings.

In conclusion this study suggests that severe depressive symptoms on admission predicts 1-year mortality in elderly patients discharged from a post-acute care unit after orthopaedic rehabilitation. Because of its potential implications, depressive symptoms should be routinely screened among elderly patients in orthopaedic rehabilitative settings.
